# Kinetic studies of few-layer graphene grown by flame deposition from the perspective of gas composition and temperature†

**DOI:** 10.1039/c9ra01257e

**Published:** 2019-07-04

**Authors:** Edhuan Ismail, Fatin Bazilah Fauzi, Mohd Ambri Mohamed, Mohd Fairus Mohd Yasin, Mohd Asyadi Azam Mohd Abid, Iskandar Idris Yaacob, Muhamad Faiz Md Din, Mohd Hanafi Ani

**Affiliations:** Department of Manufacturing and Materials, Kulliyyah of Engineering, International Islamic University Malaysia P. O. Box 10 50728 Kuala Lumpur Malaysia mhanafi@iium.edu.my; Institute of Microengineering and Nanoelectronic, Universiti Kebangsaan Malaysia 43600 Bangi Malaysia; High Speed Reacting Flow Laboratory (HiREF), Universiti Teknologi Malaysia 81310 Johor Bahru Malaysia; Faculty of Manufacturing Engineering, Universiti Teknikal Malaysia Melaka (UTeM) Hang Tuah Jaya, Durian Tunggal 76100 Melaka Malaysia; Department of Electrical and Electronic, Faculty of Engineering, National Defence University of Malaysia Kem Sungai Besi Kuala Lumpur Malaysia

## Abstract

Studies on depositions of chemical vapour deposition (CVD) diamond films have shown that flame combustion has the highest deposition rates without involving microwave plasma and direct current arc. Thus, here we report on our study of few-layer graphene grown by flame deposition. A horizontal CVD reactor was modified for the synthesis of flame deposition of few-layer graphene on a Cu substrate. It was found that graphene obtained has comparable quality to that obtained with other flame deposition setups reported in the literature as determined from Raman spectroscopy, sheet resistance, and transmission electron microscopy. Calculation of the chemical kinetics reveals a gas phase species that has a close correlation to the growth rate of graphene. This was further correlated with van't Hoff analysis of the reaction, which shows that the growth reaction has a single dominating mechanism for temperatures in the range of 400 °C to 1000 °C. Arrhenius analysis also was found to be in good agreement with this result. This study shows few-layer graphene growth proceeds through different pathways from a CVD grown graphene and also highlights flame deposition as a viable method for graphene growth.

## Abstract 2

Growth kinetics of few-layer graphene grown by flame deposition correlates to BIN20J species in a methane to soot mechanism model.

## Graphical abstract

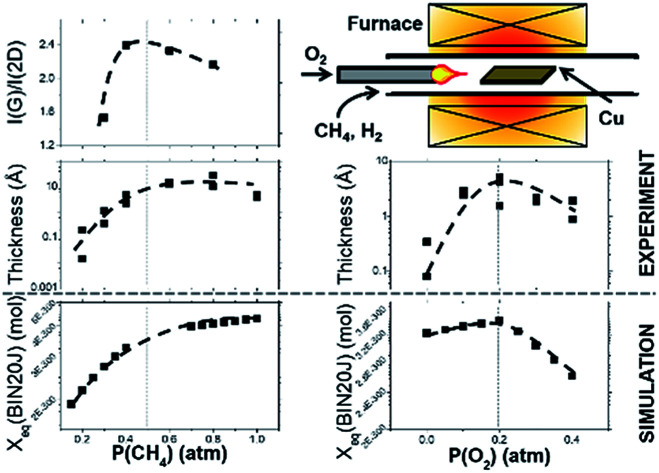

## Introduction

Current production of high-quality large-area graphene can only be achieved through chemical vapour deposition. No other method comes close to producing the quality of graphene produced by this method.^1^ However, some significant barriers remain for the widespread production of graphene by this method. One of them is that due to its batch production coupled with long reaction times at high temperatures, CVD becomes a very energy intensive method equating to increased costs. Through this method, graphene with resistivity as low as 143 Ω cm has been achieved.^2^ Bae *et al.*^3^ growing graphene films reports a sheet resistance of 30 Ω per square. In comparison, pristine graphene obtained from exfoliation of graphite has 10^−6^ Ω cm resistivity^4^ with a maximum current density^5^ larger than 10^8^ A cm^−2^.

So far, much progress has been made to reduce its energy consumption through the development of better catalysts and growth methods. In general, copper has been the choice substrate for graphene growth due to its wide availability. When compared to other elements, it is cheaper compared to other metals but its high energy barrier creates a need for high reaction temperatures for graphene to grow.^6^ Most recently, gallium and cobalt–copper alloy substrates were shown to grow graphene at lower temperatures.^7,8^

While such catalytic substrates could potentially reduce the reaction temperature and improve reaction rate, there is still a need for better production methods as it will ultimately determine the scale of which graphene can be produced. For now, modifications to the conventional CVD methods have been shown to allow good quality graphene to be produced at even lower temperatures. Usage of plasma has been widely reported to have reduced the growth temperatures of graphene and commercially manufactured reactors are already available for plasma enhanced CVD (PECVD). Instead of thermally activating the reactants within the gas phase or on the substrate, reactants are first turned to active species within the plasma itself. As a result, growth temperature could be lowered as low as <420 °C while maintaining a high-quality growth.^9,10^ However, the need for a high-power plasma generator and a good vacuum system makes the setup very complex and has high energy consumption. Thus, graphene grown by this method is very expensive due to its batch production capabilities and very strict control of the growth conditions.

Here, we explore an alternative way which could emulate plasma enhanced CVD using flame deposition. Historically, deposition of diamond by flame combustion have been widely studied and it has been shown to have a very high deposition rate when compared to other methods except for direct current arc.^11^ Previous to graphene synthesis by flame deposition, Wal *et al.* reports of the growth of carbon nanotubes by flame deposition only requiring reaction times in the order of milliseconds.^12^ The combustion itself raises the temperature allowing the remaining hydrocarbons to form the desired product. One of the earliest reports of graphene grown by flame deposition used a dual flame setup.^13,14^ Nickel substrate was kept within a flame sheath to prevent oxidation while another carburizing flame heats and provides carbon to the substrate. The authors reported that graphene synthesized through this method did not have full coverage and contains many defects. Another group built a micro combustor for the flame deposition of graphene on Cu and Ni wires where they found high Reynolds number yields better graphene qualities.^15^ A setup developed by Memon *et al.*^16^ for large area graphene growth used an inverse diffusion flame. The inverse diffusion flame getters excess O_2_ and provides a stable flame. They reported a graphene deposit which has a monolayer ratio (*I*_2D_/*I*_G_) of 0.77 to 0.59 and defect ratio (*I*_D_/*I*_G_) of 0.35 grown at 1000 °C for 10 minutes. Our group have also reported on achieving similar quality graphene using a horizontal CVD reactor modified for flame deposition.^17^ This highlights the potential that this method possesses for producing graphene at lowered temperatures.

While there are many discussions on the growth mechanism for CVD grown graphene,^18^ there are no known studies relating to growth mechanisms for graphene grown by flame deposition. However, combustion reactions of hydrocarbon have been studied in relation to diamond film formation.^19,20^ In fact, there are various models that have been built which even allows predictions of the resulting equilibrium species. In this study, we aim to study the growth kinetics of graphene grown on Cu substrate using a flame deposition method. Complimenting the experimental results are simulations of equilibrium species based upon a soot production model.^21^

## Experimental

### Deposition

**Fig. 1:**
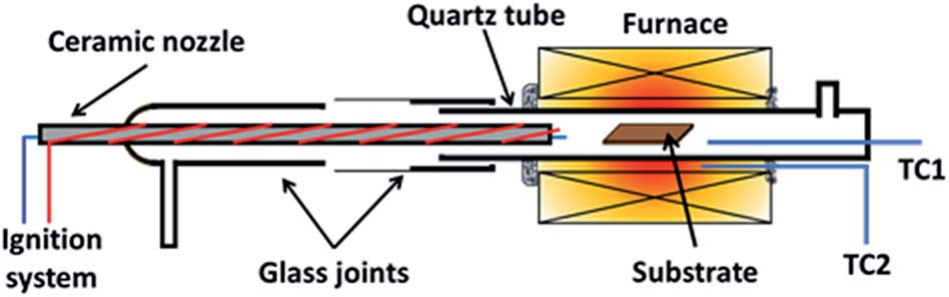
Illustration of reactor used for flame deposition made from a quartz tube with glass flanges with a ceramic nozzle.

Graphene was grown using a homemade horizontal hot-wall furnace fitted with a ceramic nozzle to allow combustion. The furnace core is a ceramic tube wrapped with Kanthal wires and insulated with ceramic fibres (Isowool, Isolite Insulating Products Co. Ltd.). An ignition system was attached to the nozzle to initiate combustion as shown in Fig. 1. A quartz tube was used as the reactor tube fitted with glass flanges at the ends. Oxygen (O_2_) gas enters from the ceramic nozzle to mix with methane (CH_4_) and hydrogen (H_2_) gas entering from a separate ingress. 2 R-type thermocouples were used to regulate and observe the temperature. One was placed with the substrate and another outside of the quartz tube within the isothermal region.

Substrate preparation before the deposition is as follows. A copper foil (0.15 mm, Magna Value Sdn. Bhd.) was polished and cleaned thoroughly before use. First, various grits of sandpaper were used until reaching #2000 grit. Next, alumina polish (1-micron MicroPolish Alumina; Buehler) was used as the final finishing before cleaning with acetone (AR grade; R&M Chemicals) followed by distilled water in an ultrasonic bath for 30 minutes. Once done, it was dried thoroughly and stored in a dry place. For the deposition, the foils were cut into 1 cm by 1.5 cm.

**Fig. 2:**
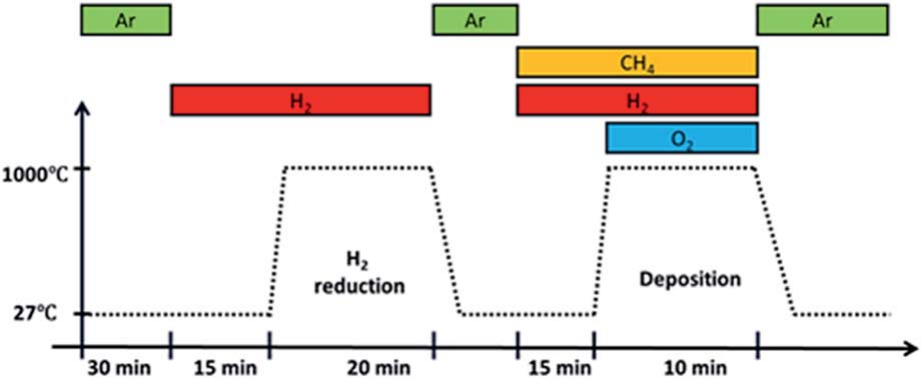
Overall procedure of growth by flame deposition. Ignition occurs upon introducing O_2_ gas into the system.

Before deposition, an annealing step was performed at 1000 °C for 20 minutes in a H_2_ atmosphere with a flow rate of 100 sccm. It was then removed from the furnace hot-zone and allowed to cool under Ar gas flow till room temperature. Once cooled, the substrate with Ar gas, CH_4_ and H_2_ were introduced followed by O_2_ gas. Upon introducing O_2_ gas, flame ignition was initiated, and the substrate was inserted back into the heated furnace at a temperature of 1000 °C for graphene deposition. After 10 minutes deposition, the substrate was taken out from the hot-zone and allowed to cool to room temperature in an Ar atmosphere. The whole reaction takes place at atmospheric pressure and its overall procedure is shown in Fig. 2.

3 parameters were tested, which are partial pressures of CH_4_, O_2_, and H_2_. Partial pressure for each reactant was changed while keeping the remaining 2 reactants at the same ratio to each other. Graphene deposits obtained were transferred and characterized.

### Transfer and characterization

Graphene deposits on Cu substrate were transferred using methyl methacrylate found commonly in super glue with tissue fibres as a reinforcing matrix. Sheet resistance measurements were done on these transferred deposits using a 4-point co-linear probe with a potentiostat (Autolab PGSTAT302N, Metrohm). Sheet resistance, *R*_s_ was then converted to deposit thickness, *t* by the following eqn (1) where graphite resistivity (*ρ* = 4 × 10^−5^ Ω cm) was used.1
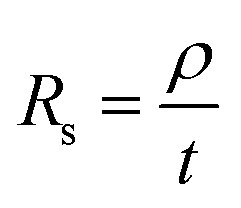


Additionally, graphene was also transferred to a silicon wafer and copper mesh grid (#300 CU TEM, U1017-5NM; EM Japan Co., Ltd.) for analysis by Raman spectroscopy (532 nm, inVia Renishaw) and transmission electron microscopy (TEM) (120 kV Talos L120C; Thermo Fischer Scientific). Fast Fourier Transform analysis was also done on bright field images from TEM using FIJI software.^22^

### Computational analysis

Apart from experimental data, numerical analysis of the concentrations of equilibrium species was also done using Cantera^23^ set to conditions similar to those stated in the experiments using a mechanism file containing 296 species.^21^

## Result and discussion

### Graphene deposition

**Fig. 3:**
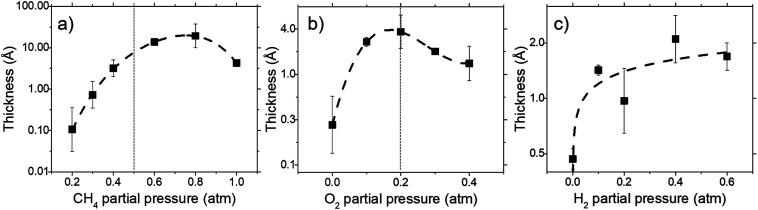
The effect of (a) CH_4_, (b) O_2_, and (c) H_2_ partial pressure on deposit thickness as measured by sheet resistance for a reaction at 1000 °C for 10 minutes. All cases maintain the remaining 2 reactant that at equal ratio to each other.

Deposit thickness calculated from the measured sheet resistance is shown in Fig. 3. From Fig. 3(a) changes in CH_4_ partial pressure, *P*(CH_4_) while H_2_/O_2_ ratio was set to unity results in thicker deposit as *P*(CH_4_) increases. However, deposit thickness reaches a maximum between 0.6 and 0.8 atm *P*(CH_4_). For the dependency of *P*(O_2_), while CH_4_/H_2_ is set to unity, deposit thickness reaches a maximum at *P*(O_2_) = 0.2 atm before decreasing again as seen in Fig. 3(b). In Fig. 3(c) it can be seen that deposit thickness increases with higher *P*(H_2_). However, it should be noted that the increase in deposit thickness due to *P*(H_2_) is very small when compared to changes due to *P*(CH_4_) and *P*(O_2_). For *P*(H_2_) deposit thickness increased from 0.5 Å to 2.1 Å whereas, graphene thickness increased by 100 fold for *P*(CH_4_) and 10 fold for *P*(O_2_). In general, conditions that yields thin deposits have a larger deviation which gets smaller as the deposits get thicker. Unsurprisingly, thinner deposits are harder to reproduce as any slight changes induces a significant change in thickness. This is clearly demonstrated in Fig. 3(a) where the error bars get smaller with thicker deposit. Since deposit obtained in Fig. 3(b) and (c) are relatively thin, deviations within the deposit thickness against partial pressure is not clear.

For Fig. 3(a), at lower *P*(CH_4_), the low initial *P*(CH_4_) coupled with high *P*(O_2_) leaves only a small amount of unreacted hydrocarbon to serve as the precursor for graphene. As it increases, there are more excess hydrocarbon allowing for thicker deposits. In Fig. 3(b) where *P*(O_2_) was varied, even a small addition of O_2_ was found to promote thicker deposits until a certain threshold which is in line with reported observations.^24^ It was noted that O_2_ was found to assist in the pyrolysis of CH_4_ which improves growth rate but higher *P*(O_2_) instead oxidizes and depletes aromatic hydrocarbons vital for the production of solid carbons.

**Fig. 4:**
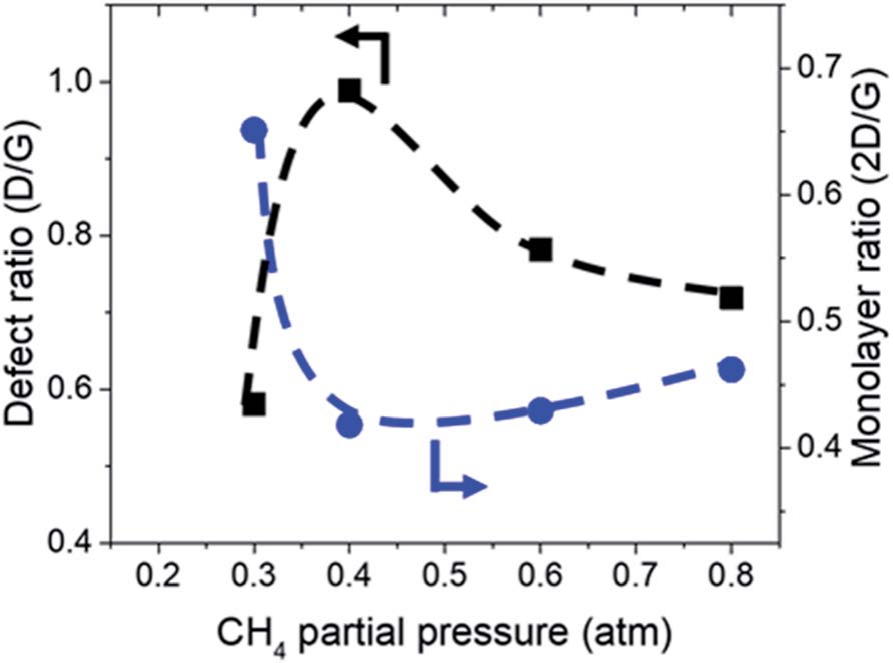
Defect ratio (*I*_D_/*I*_G_) and monolayer ratio (*I*_2D_/*I*_G_) of graphene deposited with varying CH_4_ partial pressure at 1000 °C for 10 minutes.

**Fig. 5:**
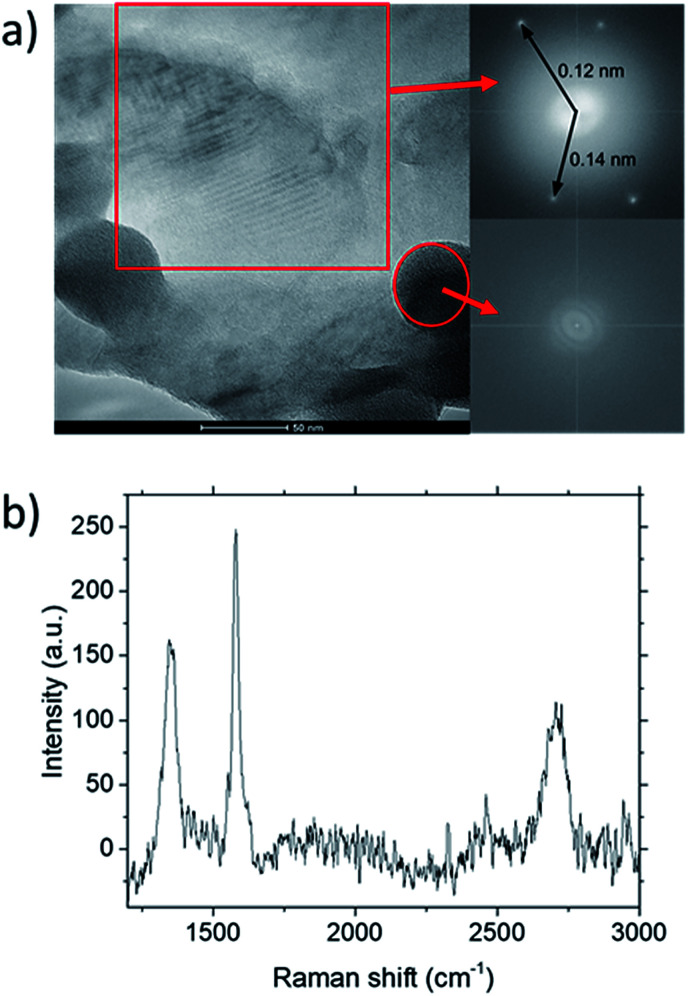
(a) TEM characterization of graphene deposited at CH_4_ : O_2_ : H_2_ = 0.8 : 0.1 : 0.1 grown at 1000 °C for 10 minutes. Inset shows FFT analysis for the highlighted regions within the image. (b) Raman spectra for the deposit.

Additionally, Raman characterization of deposits for the dependency on CH_4_ partial pressure was done and TEM analysis was performed on the deposit grown at CH_4_ : O_2_ : H_2_ = 0.8 : 0.1 : 0.1 atm which is shown in Fig. 4 and 5. Defect ratio (*I*_D_/*I*_G_) and monolayer ratio (*I*_2D_/*I*_G_) in Fig. 4 shows that higher *P*(CH_4_) yield deposits that are higher in defects and thicker which is in good agreement with thickness calculated from sheet resistance Fig. 3(a). Thus, good quality graphene could be obtained but at the cost of higher *P*(CH_4_). Again, a transition point was observed at the 0.4 atm to 0.5 atm range similar to the one in sheet resistance where thickness growth rate undergoes a change. Growth rate slows down beyond this point as O_2_ and H_2_ get further diluted by CH_4_ which hints of an optimal O_2_ and H_2_ composition in aiding the growth of graphene.

TEM analysis of the deposit reveals the presence of graphene structures and amorphous carbon. Fast Fourier Transformation (FFT) of a region with moiré pattern in Fig. 5(a) shows that it has a lattice constant of 1.2–1.4 Å which originated from the lattice of graphene.^25^ Observing another region with spherical carbon nodules yields an FFT image with rings and devoid of any spots. Raman spectra of the same deposit in Fig. 5(b) shows defect ratio higher than 0.6 and monolayer ratio of about 0.5. Several additional weak peaks were also observed. The peak at 2325 cm^−1^ was detected even on blank Cu (Fig. S1†) without graphene deposit leading us to conclude it is not carbon related. Peaks at 2640 cm^−1^ and 2946 cm^−1^ have been identified as an overtone to fundamental modes as was determined by Raman spectroscopy of highly ordered pyrolyzed graphite.^26^ Observations of deposits grown at other *P*(CH_4_) values also shows few-layer graphene as observed by Raman analysis in Fig. 4. Thus, it can be concluded that graphitic and amorphous carbon are both present in the deposit.

**Fig. 6:**
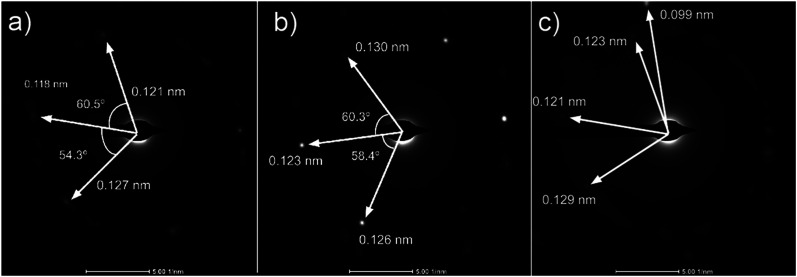
(a)–(c) Diffraction patterns from several spots of graphene deposited using CH_4_ : O_2_ : H_2_ = 0.8 : 0.1 : 0.1 at 1000 °C for 10 minutes.

Analysis of diffraction patterns of the bright spots in Fig. 6(a) and (b) shows a 6-fold symmetry. However, the angles between some spots deviate from the typical 60° which suggest the presence of differently oriented lattice. The presence of many diffraction spots forming a ring suggests the polycrystalline nature of the graphene deposited. 4 bright diffraction spots were observed in Fig. 6(b) which might be due to the uneven deposit surface. The corresponding lattice spacing calculated from these reciprocal lattices were approximately 0.12 nm. This value fits with the 0.12 nm obtained from FFT images which corresponds to the graphene's lattice spacing of 0.121 nm. In Fig. 6(c), another lattice constant of 0.099 nm was also observed, probably due to the presence of diamond-like carbons.^27^ The absence of *d* = 0.213 nm diffraction spots by graphene indicates that the deposit is few-layer graphene.^25^ Transferring the graphene on a Cu mesh introduces additional crumples and folds which will lead to regions of multilayered polycrystalline graphene which could be concluded from the twisted diffraction rings. Such defects are not originally present on the deposit itself. Overall, we could conclude that the deposit obtained contains amorphous carbon, multilayer polycrystalline graphene as well as diamond-like carbon.

### Chemical equilibrium analysis

**Fig. 7:**
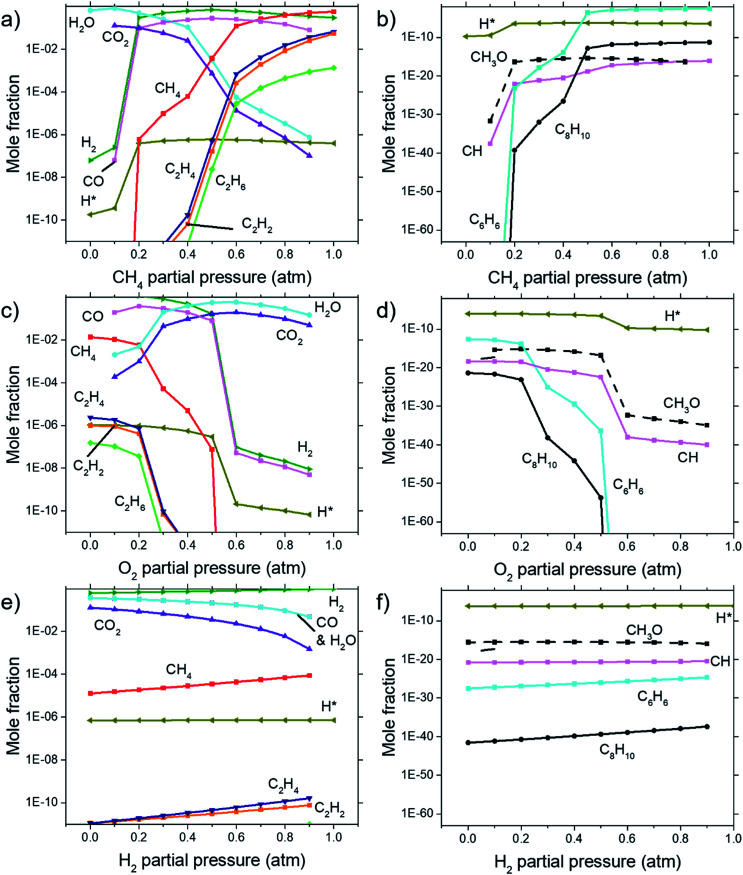
Concentrations of equilibrium species as calculated by Cantera for dependencies on (a) & (b) CH_4_ partial pressure, (c) & (d) O_2_ partial pressure, and (e) & (f) H_2_ partial pressure. Calculations were performed at 1000 °C at 1 atm. (b), (d), and (f) shows species with lower concentrations not shown in (a), (c), and (e).

Calculations for the concentration of equilibrium species for each dependency on CH_4_, O_2_, and H_2_ were performed using Cantera. Focussing on the effects of changing *P*(CH_4_), Fig. 7(a) and (b) shows that combustion products of hydrocarbon such as H_2_O, CO_2_, CO, and CH_3_O reach a peak at around *P*(CH_4_) = 0.5 atm and declines with higher *P*(CH_4_) since there is less O_2_ available. Formation of complex hydrocarbons (C_6_H_6_ and C_8_H_10_), H_2_ and H* radical undergoes a sharp increase as it transitions from a fuel lean mixture to a fuel rich mixture at *P*(CH_4_) = 0.2 atm. At higher concentrations, hydrocarbon species also increases but slow down after about *P*(CH_4_) = 0.5 atm. The growth rate of graphene observed in Fig. 3(a) and 4 also mimics this trend.

Comparisons between dependency of graphene thickness on *P*(O_2_) and its equilibrium species concentration do not present any notable similarities. However, as expected, Fig. 7(c) and (d) shows a drastic reduction of hydrocarbon and hydrogen species at *P*(O_2_) > 0.5 atm as the gas composition turns into a fuel-lean mixture. Also, the point *P*(O_2_) = 0.2 atm where deposit thickness was highest was also the point where most hydrocarbons undergo a sharp decline in equilibrium concentration as O_2_ increases. Judging from the increased concentration of combustion products, oxidation reactions are the cause for this. Whereas according to Fig. 7(e) and (f), concentrations of hydrocarbon species increases at higher *P*(H_2_) even as initial CH_4_ gets reduced due to Le Chatelier's principle to counter the increased *P*(H_2_). Deposit thickness in Fig. 3(c) was also shown to increase at higher *P*(H_2_). Regardless, equilibrium species considered here does not closely follow the changes in deposition thickness from experiments. It is probable that there are other species that have a better correlation to our experimental data.

**Fig. 8:**
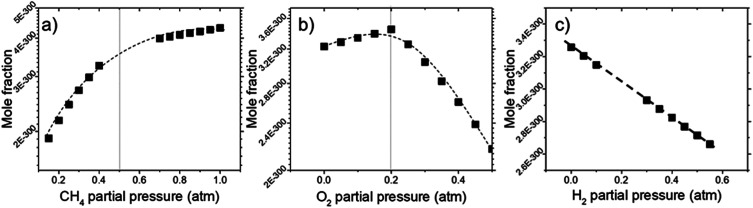
Concentrations of equilibrium species for BIN20J as calculated by Cantera for dependencies on (a) CH_4_, (b) O_2_, and (c) H_2_ partial pressure. Calculations were performed at 1000 °C at 1 atm.

In order to find the chemical species vital for graphene growth, each species available in the model was evaluated. From all the species contained within the mechanism, it was found that a chemical species C_12972032_H_1622016_ (codename: BIN20J) is a possible candidate as it possesses the best correlation to our experimentally obtained data as shown in Fig. 8. Judging from the H/C atomic ratio of 0.125 for this species, we believe this gives the closest approximation available in the model to the deposit thickness obtained experimentally.

The equilibrium concentration for BIN20J displays a direct correlation to deposit thickness for *P*(CH_4_) and *P*(O_2_) in Fig. 8(a) and (b). Analysis of reaction order shown in Fig. S2† also shows that growth rate is a 1^st^ order reaction which explains the close correlation of BIN20J concentration to deposit thickness. Calculations on the yield and selectivity of BIN20J was also done (Fig. S3†), it shows decreasing yield and selectivity as *P*(CH_4_) increases as more hydrocarbon by-products are also produced at higher methane concentration. Selectivity for BIN20J increases above *P*(O_2_) = 0.2 atm but its yield reaches a plateau at that point. This is believed to be due to its consumption by excess oxygen despite its increased selectivity. Changes to deposit thickness in relation to *P*(H_2_) shows poor correlation to the predicted values of BIN20J. However as noted previously, changes in deposit thickness due to H_2_ dependency were an order of magnitude smaller than those due to CH_4_ and O_2_. Studies have noted that graphene could still be obtained by flame deposition even without H_2_ gas being used^28^ and chemisorbed hydrogen on Cu functions as a catalyst for CH_4_ heterogenous dissociation on Cu.^29^ From this, we believe that H_2_ has a small influence in gas phase kinetics and instead has a bigger role in affecting the substrate itself directly. Since the model involves only gas phase reactions, such heterogeneous reactions are left unaccounted for thus leading to the inconsistency between experimental and predicted model. Regardless, the predicted values of BIN20J has been shown to have a good correlation to experimental data.

The close correlation found between experimental values of deposit thickness and equilibrium concentration of BIN20J was further evaluated by Arrhenius and van't Hoff analysis. Both are useful tools in understanding reaction kinetics.^30^ An Arrhenius plot (eqn (2)) of rate constant, *k* against the temperature reciprocal, 1/*T* as well as equilibrium constant, *K*_eq_ against 1/*T* of the van't Hoff equation (eqn (3)) gives useful information on the reaction mechanism.2
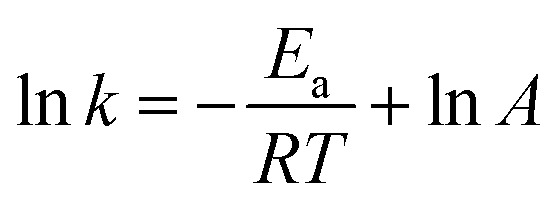
*k*: reaction rate constant, *E*_a_: activation energy, *R*: gas constant, *T*: temperature, *A*: pre-exponential factor.3
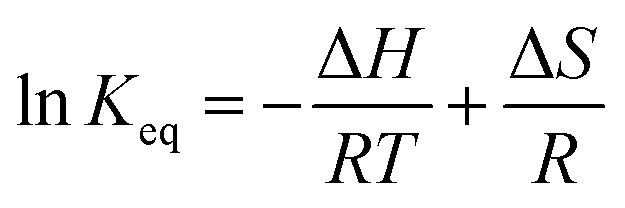
*K*_eq_: equilibrium constant, Δ*H*: enthalpy energy, *R*: gas constant, *T*: temperature, Δ*S*: entropy energy.

**Fig. 9:**
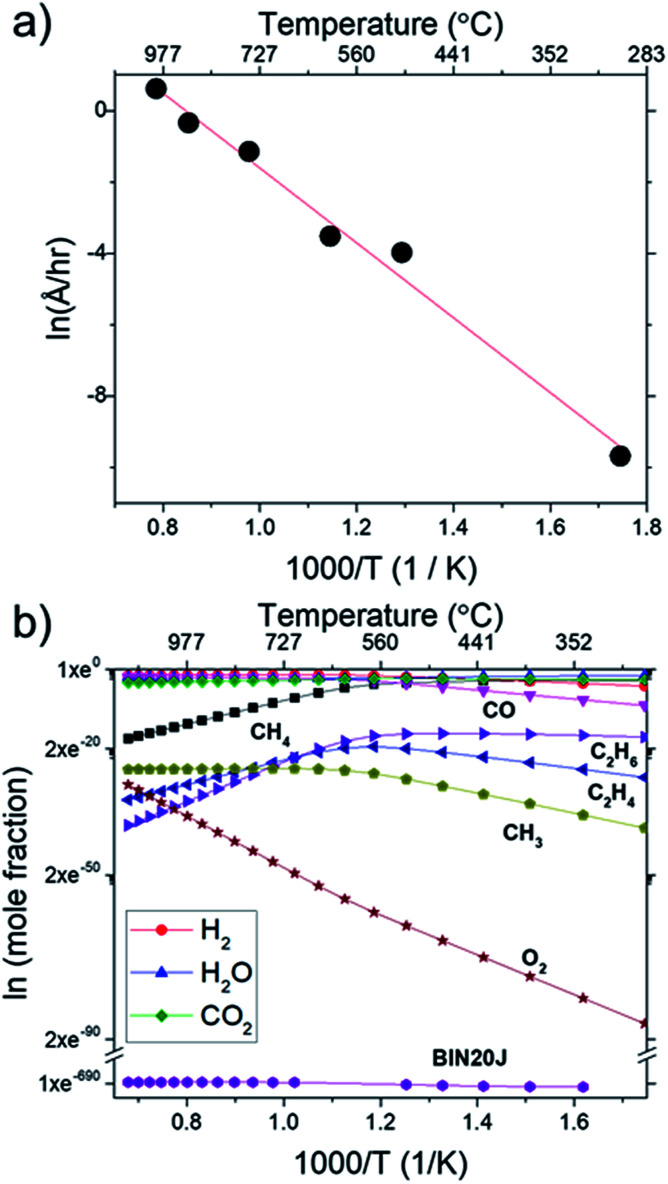
(a) Arrhenius plot for graphene growth rate calculated from sheet resistance and (b) van't Hoff plot of various species as calculated by Cantera. Calculations were performed at 1000 °C at 1 atm.

In Fig. 9(a), Arrhenius plot of graphene growth rate shows that a single mechanism dominates across the temperature range of 300 °C to 1000 °C. The van't Hoff plot in Fig. 9(b), however, shows that only BIN20J has a single reaction mechanism responsible for its production within the same temperature range. Other species have 2 distinct kinetic regimes involved in its production which has a transition at around 600 °C. As an example, formation of C_2_H_4_ was shown to be endothermic at lower temperatures but exothermic at higher temperatures indicating a change in reaction mechanism. Experimentally, gas phase analysis during methane combustion also shows this mechanism transition^31^ which was corroborated by numerical analysis.^32^ The transition as temperature was increased beyond 600 °C was attributed to the mass transport as being the limiting step at such accelerated reaction rates. The fact that BIN20J remains linear could be due to it being a heavier hydrocarbon (*i.e.* soot particles) that is less affected to the gas phase kinetics.

The calculated activation energy of our deposition reaction was 0.9 eV (87 kJ mol^−1^) which is markedly lower than graphene grown by conventional CVD methods as seen in Table 1, #1. In a typical LPCVD, the rate-limiting step for graphene was determined to be the dissociation of adsorbed CH_4_ as determined by *ab initio* studies^33^ and mass transport is responsible for the rate-limiting step at APCVD^34,35^ which generally leads to an increased energy barrier (Table 1, #1–#7). Only through the use of PECVD, activation energy lower than our flame deposition method was achievable (Table 1, #8). Also, most reported activation energies for conventionally grown CVD graphene has 2 separate *E*_a_ which infers 2 separate kinetic regimes for graphene growth (Table 1, #1). Additionally, CH_4_ combustion was also reported to have 2 distinct mechanisms, a slow reaction and ignition depending on the temperature.^36,37^ Also, due to the nature of this method being of a combustion reaction, soot production (Table 1, #9) and diamond formation (Table 1, #10) was also considered but it was found to have very different activation energies. In contrast, flame deposition only shows a single mechanism from the Arrhenius plot which means graphene grows by a different mechanism path which is also unique from diamond and soot formation.

**Table 1:** Reported activation energies of graphene growth and related reactions

Reaction	Temperature/°C	Activation energy/eV	Note
**Growth rate**	**300–1000**	**0.9 (87 kJ mol** ^**−1**^ **)**	**Flame deposition (this study)**
(1) Nucleation density	900–1000	3	Cu LPCVD^38^
	750–850	1	
(2) Nucleation density	950–1050	4	Cu LPCVD^39^
(3) Growth rate	935–1010	4.5	Cu LPCVD^40^
(4) Growth rate	907–987	2.7	Cu LPCVD^41^
(5) Nucleation density	950–1050	9	Cu APCVD^39^
(6) Growth rate		5	
(7) Growth rate	900–1050	2.74	Cu APCVD^42^
(8) Growth rate	600–800	0.57	SiO_2_ PECVD^43^
(9) Soot growth	1949–2167	2.1	ref. 24
(10) Diamond growth	n.a.	2.38	ref. 44

While a thorough understanding of the growth mechanism of graphene by flame deposition still eludes us, our results provides certain insights on the growth of graphene by flame deposition. First, graphene growth follows closely with the BIN20J formation using a soot formation mechanism reported by Ergut *et al.*^21^ In this case, BIN20J formation in the gas phase begins from decomposition of CH_4_ into CH_3_ which then undergoes various recombination reaction to form heavier hydrocarbon leading to BIN20J. Second, the big difference in reported activation energies of graphene growth by CVD eliminates such mechanism that involves heterogeneous decomposition of CH_4_ on Cu and those of soot or diamond formation. From the formation of BIN20J, it will then adsorb on Cu substrate to provide a nucleation point for graphene growth. At this point, how BIN20J develops into graphene is unclear but we believe that it might proceed through CH_4_ decomposition promoted by adsorbed oxygen since it possesses activation energy (0.9 eV) closest to our experimental values.^45,46^

## Conclusions

An experimental flame deposition setup has been built for the growth of few-layer graphene. Raman analysis shows multilayer graphene with a monolayer ratio (*I*_2D_/*I*_G_) of 0.65 and defect ratio (*I*_D_/*I*_G_) of 0.58 which was comparable to literature. We also show that numerical analysis by an open source software using an appropriate mechanism model provides a close prediction to the actual graphene deposition. Changes to deposit thickness when CH_4_ and O_2_ were varied was found to agree with simulated values for BIN20J. Deviations between experimental and simulation results for *P*(H_2_) suggests H_2_ has a minor role in gas phase kinetics and influences the heterogeneous reaction on the substrate which is not accounted for by the mechanism file used. At this current stage only the gas phase mechanism was considered but with proper improvements, a model that could predict the actual deposition is certainly possible. Considerations of the growth kinetics lead us to believe that the growth mechanism includes the gas phase formation of BIN20J followed by its adsorption on Cu substrate which then grows into graphene.

## Conflicts of interest

There are no conflicts to declare.

## Supplementary Material

RA-009-C9RA01257E-s001
