# Supplementary material for: Kinetic studies of few-layer graphene grown by flame deposition from the perspective of gas composition and temperature
Source: RSC Adv. 2019 Jul 4;9(36):21000–8. doi: 10.1039/c9ra01257e (PMC9065698; doi:10.1039/c9ra01257e)
Supplement: RA-009-C9RA01257E-s001 [file RA-009-C9RA01257E-s001.pdf]

Electronic Supplementary Material (ESI) for RSC Advances.
This journal is © The Royal Society of Chemistry 2019

Supporting Information

Kinetic Studies of Few-Layer Graphene Grown by Flame Deposition from the Perspective of Gas Composition and Temperature

Edhuan Ismail[a], Fatin Bazilah Fauzi[a], Mohd Ambri Mohamed[b], Mohd Fairus Mohd Yasin[c], Mohd Asyadi Azam Mohd Abid[d], Iskandar Idris Yaacob[a], Muhamad Faiz Md Din[e], Mohd Hanafi Ani[a]*

[a] Department of Manufacturing and Materials, Kulliyyah of Engineering, International Islamic University Malaysia, P.O. Box 10, 50728 Kuala Lumpur, Malaysia
[b] Institute of Microengineering and Nanoelectronic, Universiti Kebangsaan Malaysia, 43600 Bangi, Malaysia
[c] High Speed Reacting Flow Laboratory (HiREF), Universiti Teknologi Malaysia, 81310 Johor Bahru, Malaysia.
[d] Faculty of Manufacturing Engineering, Universiti Teknikal Malaysia Melaka (UTeM), Hang Tuah Jaya, Durian Tunggal, 76100 Melaka, Malaysia
[e] Department of Electrical and Electronic, Faculty of Engineering, National Defence University of Malaysia, Kem Sungai Besi, Kuala Lumpur, Malaysia

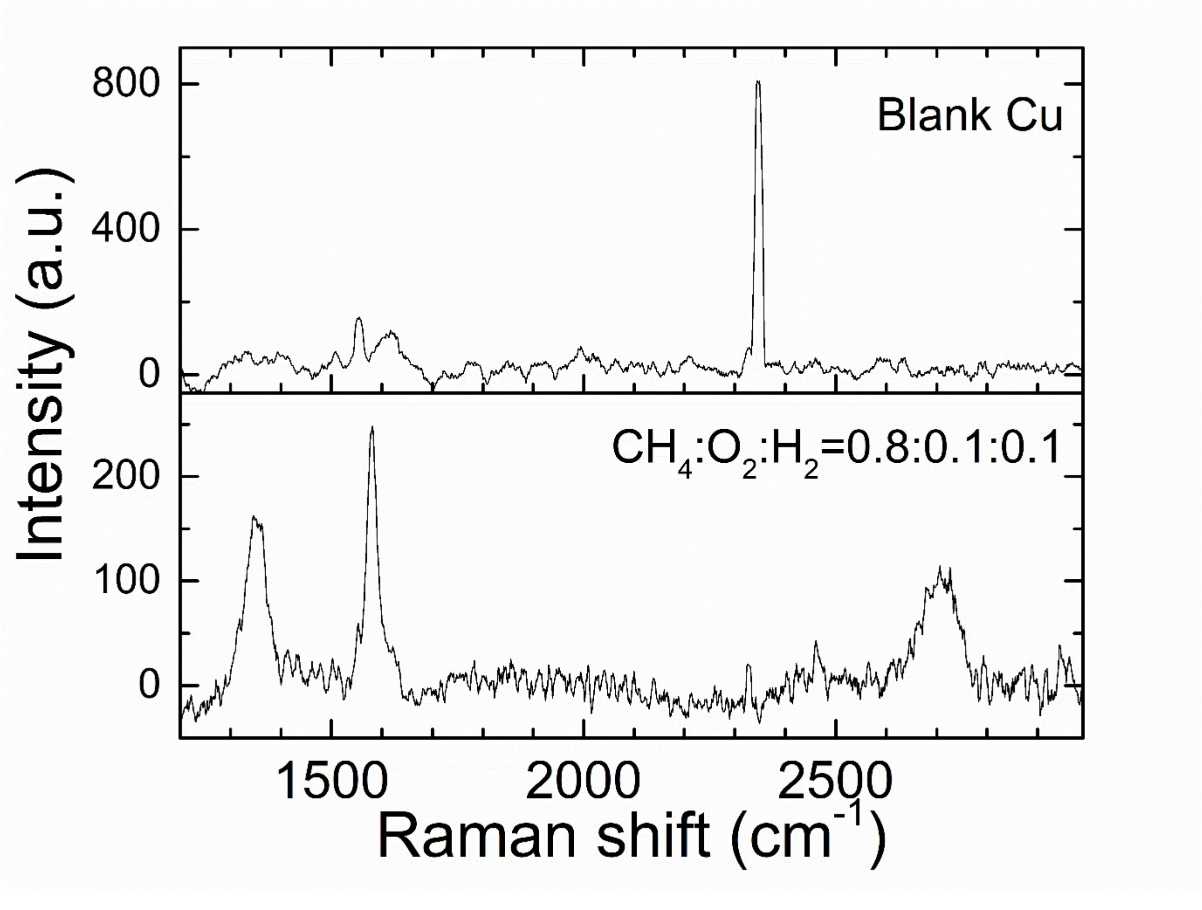

Figure S1- Comparison of Raman spectra between blank Cu and graphene on Cu substrate grown at 1000°C for 10 min using CH_4:O_2:H_2=0.8:0.1:0.1.

The Raman spectroscopy machine used consistently observed a very large peak at 2330 cm^{-1} even when no graphene was produced as can be seen from the blank Cu spectra in Figure S1. Its cause could not be identified. However, graphitic peaks could still be detected despite its presence and thus it is removed in consequent spectra for ease of analysis. Apart from that, many additional peaks were

observed for samples with graphene deposits but due to it being very small in contrast to graphitic peaks (D, G and 2D bands), it was assumed to be noise related and ignored during characterization.

Calculations for the yield and selectivity of BIN20J has been performed. As an initiation step, methane was assumed to first decompose into its activated state, CH_3 radicals before undergoing any reactions[1]. Thus, methane conversion rate is the amount of CH_3 species against the initial CH_4 concentration (Eq. S1). Selectivity is the ratio of BIN20J that forms from this CH_3 species (Eq. S2) and its yield is its ratio against the initial methane concentration (Eq. S3).

$$Conversion = [CH_3]/[CH_4]_{initial} \quad (S1)$$
$$Selectivity = [BIN20J]/[CH_3] \quad (S2)$$
$$Yield = [BIN20J]/[CH_4]_{initial} \quad (S3)$$

In relation to the graphene growth to BIN20J species, studies on the reaction order of graphene has been performed as shown in Figure S2. Growth rate was determined to be likely 1st order reaction meaning that a single species is mostly responsible for the growth rate, *v*. Here, we believe that BIN20J is the species that is responsible for graphene growth as its predicted changes in concentration has good correlation to graphene growth. This holds true for when CH_4 or O_2 partial pressure was tested as was discussed in the main article shown again here in Figure S3 (a-f). Thus, growth rate, *v* could be represented as Eq. S4.

$$Graphene\ growth\ rate,\ v \propto k[BIN20J] \quad (S4)$$

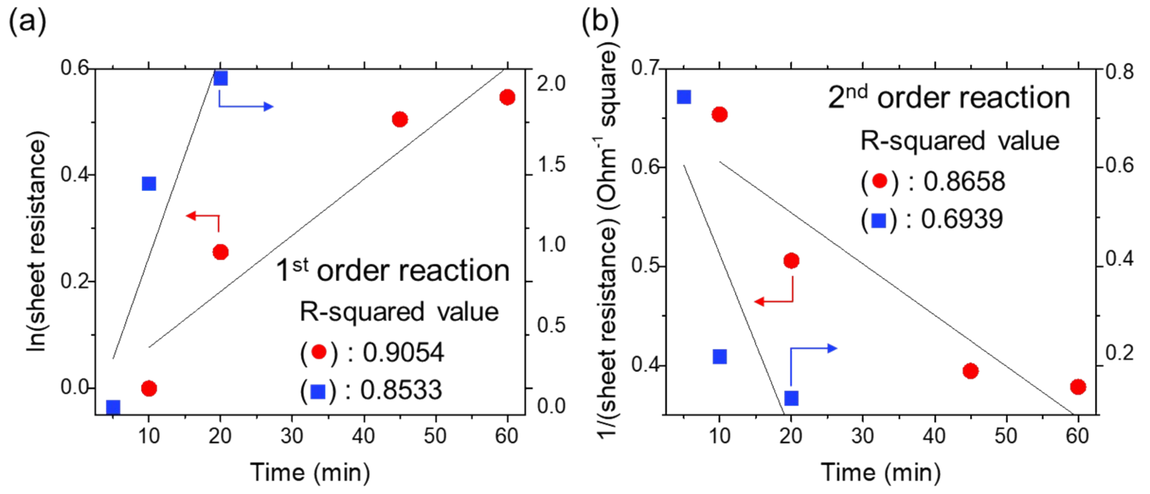

Figure S2- Fitting of sheet resistance (graphene growth rate) to a 1st order reaction rate and 2nd order reaction rate. Two sets of reactions were performed. For the red circle (●), gas composition was CH_4:O_2:H_2=20:20:30 sccm and temperature was 1000°C and blue square (■) was CH_4:O_2:H_2=30:50:20 sccm and 750°C.

Figure S3 (b, e, and h) shows that calculations of yield and selectivity shows that only BIN20J yield during O_2 partial pressure studies matches growth rate. Changes in CH_4 and H_2 partial pressure display a poor correlation between predicted yield to experimentally determined growth rate (Figure S3). For the case of H_2, as per the discussion in the main article, it is believed to have a minimal role

in graphene growth in flame deposition conditions. For the case of CH_4, a large increase in initial CH_4 concentration only leads to additional hydrocarbon by-products with only reduces the yield and selectivity of BIN20J. Figure 7 (a) & (b) shows that a large amount of hydrocarbons being produced and these species has been determined to have no direct correlation to changes in deposit thickness (i.e. graphene growth) at all. Presumably, O_2 gas role in the elimination of all hydrocarbons when in excess leads to a yield trend that is close to the changes in deposit thickness. Despite so, Figure S3 (h) shows that BIN20J was selectively produced at higher O_2 partial pressure. This might be that since, BIN20J is a supramolecule that is similar to graphene and thus possesses a certain resistance to chemical attacks compared to simpler hydrocarbon by-products.

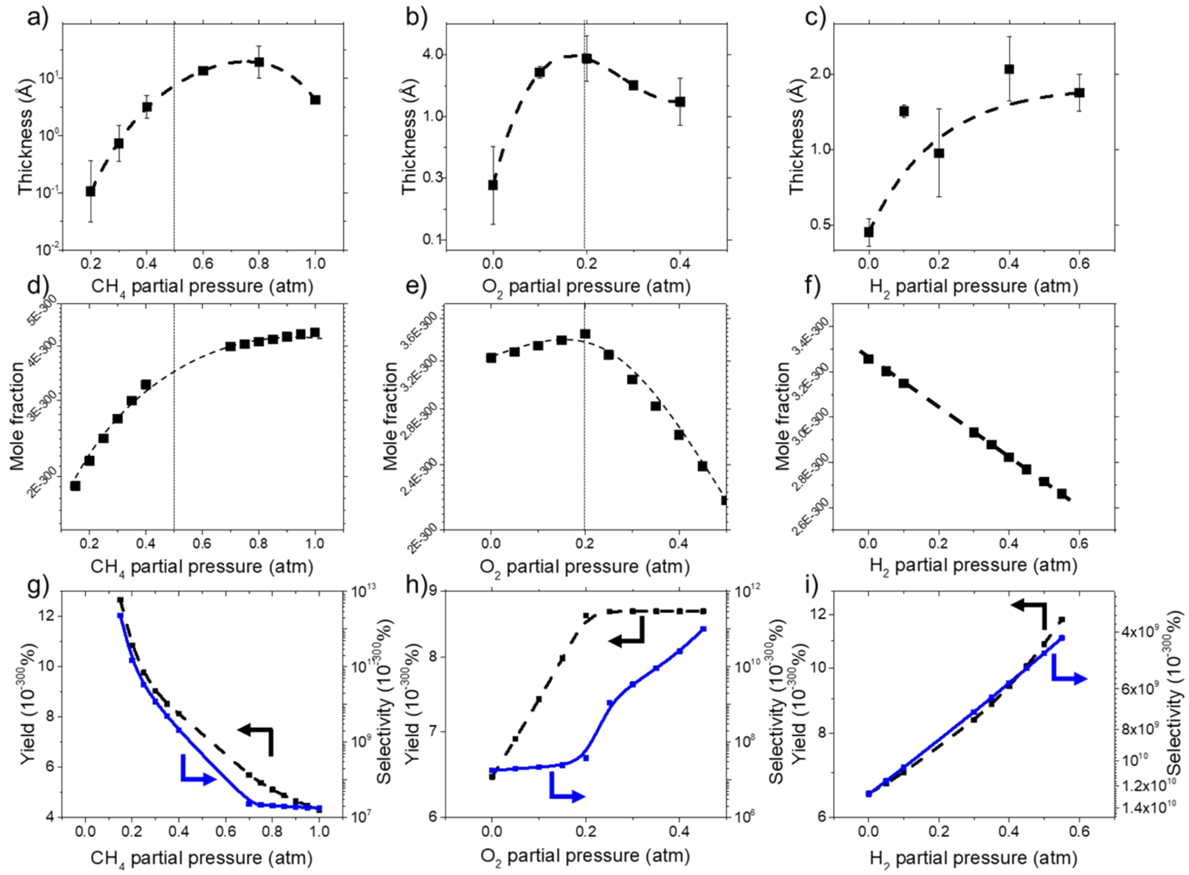

Figure S3- (a-c) Experimentally measured deposit thickness of graphene grown at 1000°C for 10 min. For each graph the remaining reactants are at 1:1 ratio. (d-f) Predicted molar fraction of BIN20J and (g-i) its calculated selectivity and yield for the same partial pressure range.

References

1 A. Holmen, O. Olsvik and O. A. Rokstad, *Fuel Process. Technol.*, 1995, **42**, 249–267.
